# Environmental Sampling for Spores of *Bacillus anthracis*

**DOI:** 10.3201/eid0810.020398

**Published:** 2002-10

**Authors:** Eyasu H. Teshale, John Painter, Gregory A. Burr, Paul Mead, Scott V. Wright, Larry F. Cseh, Ronald Zabrocki, Rick Collins, Kathy A. Kelley, James L. Hadler, David L. Swerdlow, members of the Connecticut Anthrax Response Team

**Affiliations:** * Centers for Disease Control and Prevention, Atlanta, Georgia, USA; † Agency for Toxic Substances and Disease Registry, Atlanta, Georgia, USA; ‡ National Institute for Occupational Safety and Health, Cincinnati, Ohio, USA; § Connecticut Department of Public Health, Hartford, Connecticut, USA.

**Keywords:** *Bacillus anthracis*, anthrax, environmental sampling, postal facility, surface sampling, HEPA vacuum sock, swabs, wipes

## Abstract

On November 11, 2001, following the bioterrorism-related anthrax attacks, the U.S. Postal Service collected samples at the Southern Connecticut Processing and Distribution Center; all samples were negative for *Bacillus anthracis*. After a patient in Connecticut died from inhalational anthrax on November 19, the center was sampled again on November 21 and 25 by using dry and wet swabs. All samples were again negative for *B. anthracis*. On November 28, guided by information from epidemiologic investigation, we sampled the site extensively with wet wipes and surface vacuum sock samples (using HEPA vacuum). Of 212 samples, 6 (3%) were positive, including one from a highly contaminated sorter. Subsequently *B. anthracis* was also detected in mail-sorting bins used for the patient’s carrier route. These results suggest cross-contaminated mail as a possible source of anthrax for the inhalational anthrax patient in Connecticut. In future such investigations, extensive sampling guided by epidemiologic data is imperative.

Following the bioterrorism-related anthrax attacks in October 2001, a total of 22 cases of anthrax were identified: 11 confirmed cases of inhalational anthrax, and 11 (7 confirmed and 4 suspected) cases of cutaneous anthrax (1). Epidemiologic investigation of the first nine patients with inhalational anthrax showed that they were exposed to particulate aerosols containing *Bacillus anthracis* when they opened letters or when letters were processed in postal facilities (2).

The final case of inhalational anthrax in 2001, reported on November 19, was in a 94-year-old woman from Oxford, Connecticut, who died (3). Unlike previous cases, the patient was not a postal employee, mail handler, media worker, or government official ( 1,2). An extensive investigation for *B. anthracis* spores was conducted at her home and other places that she visited in the 2 months preceding her death; all samples were negative (4). Retrospective and prospective surveillance detected no additional cases of anthrax in her community (5,6), and an intentional release of anthrax spores there was considered unlikely. The investigation focused on mail as the source of anthrax; we subsequently conducted intensive sampling of the postal facility that serves her region. We describe the sampling methods, results, and public health implications of repeated environmental sampling in this facility.

## The Setting

The regional postal processing center for the patient is the Southern Connecticut Processing and Distribution Center (SCPDC) in Wallingford. With a floor area of 350,000 square feet and the capacity to process up to 3 million pieces of mail a day, the center is in operation around the clock. In November 2001, SCPDC employed 1,122 workers.

The center is equipped with 6 advanced-facer canceller machines, 5 optical character reader machines, 5 bar-code sorting machines, and 13 digital bar-code sorting (DBCS) machines for processing letters. In addition, automated flat sorting machines, linear integrated parcel sorters, and small bundle and parcel sorters are used to process flats (large flat mail that is not a package) and parcels (wrapped packages). Although all these machines are part of the facility, they differ in function, speed of processing, and location within the facility.

### Mail Processing

The advanced facer-canceller machines cancel letters originating from southern Connecticut and apply two bar codes that are used to identify and sort letters for their final destination. The identification tag, a fluorescent orange bar code on the back of the envelope, records the time and date that the letter was canceled. The postnet barcode, a series of vertical full and half bars applied to the front of an envelope, contains zip code and delivery point information in machine-readable format. Advanced facer-canceller machines are used primarily to process stamped mail; bulk letters are not processed on canceling machines because they already have barcodes applied by the mailers and are presorted.

The high-speed computerized DBCS machines are used for preliminary and final sorting of the mail by barcode. During the preliminary sort, letters can be processed on any one of the 13 DBCS machines at the facility. This step arranges the letters by the 5-digit zip code of the delivery address, usually requiring <2 passes to sort a batch of mail. Once this step is accomplished, mail is transported for final processing to a designated DBCS machine, which sorts the letters to the 9- or 11-digit zip code, usually requiring <3 passes. Therefore, letters addressed to the patient could have been processed initially on any of the 13 DBCS machines. Later, the final sort would have been processed on DBCS no. 6, and specific bins were designated for the carrier route.

In October and November 2001, independent contractors working for the U.S. Postal Service (USPS) tested postal processing and distribution plants nationwide to determine if any had become contaminated with *B. anthraci*s following the bioterrorism events. As part of this effort, SCPDC was tested on November 11, 2001; all results were negative for *B. anthracis* contamination. Following the report of the inhalational anthrax case in Oxford, Connecticut, the facility was tested again extensively.

## Methods

Samples were obtained from SCPDC on November 11, 21, 25, and 28 and December 2. Sampling methods included dry swabs, wet swabs, wet wipes, and HEPA vacuum.

On November 11, a contracting company working for USPS obtained samples from SCPDC as part of the nationwide testing of postal facilities for anthrax spores. The contractor took dry synthetic swabs from random sites in the facility and sent them to be analyzed at the Connecticut Department of Public Health Laboratory. On November 21, 2001, after the report of the 94-year-old woman with anthrax in Connecticut, a second independent contractor hired by USPS collected additional dry swab samples from surfaces where letters, flats, and parcels were processed. These samples, along with others collected from air circulating units, were analyzed by the Connecticut Department of Public Health Laboratory.

On November 25, the investigation team obtained samples from the facility using wet synthetic swabs and processed them by methods recommended by CDC (7,8). Samples were taken from the letter canceling and sorting machines, flat and parcel sorting machines, and five facility vacuum filters in use since October 27, 2001. The samples were analyzed by the Connecticut Department of Public Health Laboratory.

Samples taken on November 28 were more extensive. Guided by additional epidemiologic data, we collected samples from carefully selected sites (the canceling and sorting machines) by using wet synthetic 2x2-inch wipes and HEPA vacuum. Specimens were collected and transported according to recommended methods (7,8). Wipe and vacuum samples were cultured and analyzed at a CDC-contracted laboratory.

On December 2, following the first report of anthrax-positive results in the facility, we collected follow-up samples. A composite sample from the vertical column of four bins was taken from all columns on the four DBCS machines that were presumptively positive based on sampling done on November 28. These wet wipe samples, taken to determine the extent of contamination on the machines, were analyzed by a CDC-contracted laboratory.

## Results

A total of 589 samples were collected from November 11 to December 2, 2001. Three hundred forty-six (59%) of these were from the DBCS machines. Of the 589 samples, 117 (20%) were dry swabs, 60 (10%) wet swabs, 300 (51%) wet wipes, and 112 (19%) HEPA vacuum samples.

Fifty-three dry synthetic swab samples were taken on November 11. Of these, only one (2%) sample was from a DBCS machine (no. 6). All samples were negative for *B. anthracis* (Tables 1,2).

**Table 1:** Number of samples taken from digital bar-code sorting machines during five sampling dates, Connecticut, 2001

Machine no.	11/11/01	11/21/01	11/25/01	11/28/01	12/02/01	Total samples
1			1	8		9
2			1	8		9
3				8		8
4				11^a^	48 ^a^	59
5		2		12		14
6	1	2	3	23	48 ^a^	77
7		2		12		14
8				8		8
9			1	8		9
10				8^b^	52^c^	60
11			1	8 ^a^	52^d^	61
12				8		8
13			1	8		9
Total	1	6	8	130	200	345

^a^One positive sample. ^b^Four positive samples. ^c^Thirty positive samples. ^d^Three positive samples.

**Table 2:** Environmental sampling methods, types, and results of samples taken November 11– December 2, Southern Connecticut Processing and Distribution Center, 2001^a^

Sampling date	No. of samples	Samples from DBCS	Type	Positive results	Sample collectors
11/11/01	53	1	Dry swabs	0	USPS
11/21/01	64	6	Dry swabs	0	USPS
11/25/01	60	8	Wet swabs	0	CDC/ATSDR
11/28/01	212	131	Wet wipes and vacuum	6	CDC/ATSDR
12/02/01	200	200	Wet wipes	35	CDC/ATSDR
Total	589	346		41	

^a^DBCS, digital bar-code sorting; USPS, United States Postal Service; CDC, Centers for Disease Control and Prevention; ATSDR, Agency for Toxic Substances and Disease Registry.

On November 21, 64 dry synthetic swab samples were taken. Of these, six (10%) were from the DBCS machines, two each from DBCS nos. 5, 6, and 7. All samples were negative for *B. anthracis* (Tables 1,2).

On November 25, the investigation team took a total of 60 wet synthetic swab samples; 8 (13%) were from the DBCS machines. Of the eight samples taken from the DBCS machines, one sample each was taken from DBCS nos. 1, 2, 9, 11, and 13 and three from DBCS no. 6. All samples were negative for *B. anthracis* (Tables 1,2).

On November 28, the most extensive sampling was conducted, with 212 samples collected. Of these, 102 (48%) were wet wipes and 110 (52%) vacuum samples. We used wet wipes for sampling the stacker bins (hard surfaces) and the HEPA vacuum for sampling the machines, including the inaccessible parts. We focused our sample collection on machines likely to have processed mail delivered to the patient's address. Although all machines were tested, 131 (62%) samples were from DBCS machines, which processed both stamped mail and nearly all the bulk presorted mail; approximately 80% of the mail recovered from the patient's home was bulk mail.

Of 212 samples, 6 (3%) yielded *B. anthracis*, and all positive samples were from DBCS machines. Of the six anthrax-positive samples, two were vacuum samples from DBCS nos. 4 and 10, and four were wet wipe samples from the bins of DBCS machines nos. 10 and 11. One vacuum sample (0.55 g of specimen) from the feeder part of machine no. 10 had 2.9x10^6^ CFU of *B. anthracis*, equal to 5.5x10^6^ CFU of *B. anthracis* per gram of sample material. Of the mail sorted on this machine, approximately 75% is bulk mail. This machine has not been sampled before November 28, the fourth round of sampling.

Following the results of the sampling on November 28, we collected follow-up samples on December 2. We took samples to determine the extent of contamination on DBCS machines nos. 4, 10, and 11, the machines from which results were positive for *B. anthracis* on the November 28 sampling. In addition, we also collected samples from DBCS machine no. 6 because preliminary positive results from the November 28 sampling were reported and because this machine was used for final processing of mail to the address of the patient. The 200 wet wipe samples taken on December 2 were composite wipes from a vertical column of four bins from each machine (each machine has 48–52 columns of four bins). We collected composite samples to allow complete sampling of all bins from all suspect machines without taking an excessive number of samples (Table 2).

Of 200 composite column samples from DBCS machines nos. 4, 6, 10, and 11, a total of 35 (17.5%) columns of bins were positive. On machine no. 10, 30 (68%) of 52 columns were positive. Three (6%) of 52 columns from machine no. 11 and 1 (2%) of 48 columns on both machines no. 4 and 6 were positive. These results confirmed the high contamination of machine no. 10. Only 1 of 48 columns of bins on machine no. 6 was found to be positive. Machine no. 6 was used for final mail sorting for several zip codes including the town where the patient lived. The only column of bins that yielded *B. anthracis* on DBCS no. 6 included bins for the carrier route for the patient’s home.

## Discussion

Supplemented by the findings of the epidemiologic investigation team, our investigation identified cross-contaminated mail as a possible source of anthrax for the Connecticut patient (4). No other source of contamination in her community was identified after extensive sampling of her home and areas she visited; no other cases of anthrax were reported. We identified a contaminated sorting machine that was used to sort most of the mail delivered to the patient, including bulk mail; the specific column of bins that held mail for her carrier route was positive (4). Extensive sampling with large numbers of samples was required to find anthrax spores. Positive results were obtained following sample collection based on information learned during the epidemiologic investigation. All positive results were obtained from samples collected by using wet wipes and vacuum sampling. All the dry or wet swab samples were negative for *B. anthracis*.

Environmental sampling during an anthrax investigation is critical in determining the likely source of infection and the extent and degree of environmental contamination, to support decisions on the need for prophylaxis with antibiotics or clean-up, and to provide guidance about when clean-up is adequate to permit reentry into an area. During this investigation, no validated methods for specifically sampling the environment for *B. anthracis* were known. We lacked data on the effectiveness of the sample collection media (swabs, wipes, and vacuum) for typical porous and nonporous surfaces encountered in indoor environments. The effect of varying concentrations of *B.* *anthracis*–containing particles and dust loading on sampling efficiency had also not been studied. Furthermore, recovery efficiency of the analytical methods (efficiency of removal of *B. anthracis* spores from the sample collection media) had not been adequately evaluated, and limits of detection have not been established (8).

Although our investigation showed that different sample collection techniques and sampling sites and numbers of samples yielded different findings, results are based on observation and cannot be used to specifically compare the different approaches. However, exploring the reasons for the different results may be useful for future investigations. On November 11, all samples were collected by using dry swabs from random sites in the facility with the intent of finding contamination anywhere in the facility. Only one sample from the DBCS machines was taken. On November 21, more samples were taken from the DBCS machines, but still only three machines were sampled. This sampling was performed with emphasis on the Oxford mail route because the illness had been reported in that community. However, whether the patient’s mail was predominantly bulk mail and whether letters could have been sorted preliminarily on any DBCS machine were not known at the time. The November 25 sampling was similar to the November 21 sampling except that investigators used wet swabs instead of dry swabs. Again, limited samples from six DBCS machines were taken.

On November 28, more extensive and directed sampling was conducted, and epidemiologic information was available to guide us to the appropriate sites. Using wet wipes and HEPA vacuum led to the first positive results for anthrax in the facility. A recent study, conducted after the Connecticut investigation, has confirmed our findings (WT Sanderson et al., unpub. data). In this study, side-by-side surface swabs, wipes, and HEPA vacuum samples were taken at the Brentwood Processing and Distribution Center in Washington, D.C., to compare their relative effectiveness in a contaminated postal facility. Wet wipes and vacuum sampling were found to be more effective methods than surface swabs; results from wet wipes and vacuum samples were highly concordant. Of 28 sample locations tested, 4 (13%) were positive with dry swabs, compared with 13 (46%) wet swabs, 23 (82%) wet wipes, and 23 (82%) vacuum samples (WT Sanderson et al., unpub. data).

Although the effectiveness of sampling techniques influences which are used, other factors that determine the choice of sampling techniques include the site of sampling, size of the surface to be sampled, character of the surface (porous or nonporous), need to quantify the results, and preference and specialization of the laboratory where the test is done. Swab samples may still be the best method to sample small hard surfaces not easily accessible for wiping or vacuum sampling (e.g., a keyboard). Surface wipes also have several limitations (8). Wipe samples might miss minimally contaminated surfaces or small, discrete contaminated areas. In addition, sampling all surfaces within a building by using surface wipes is not feasible. Therefore, vacuum samples provide an important tool for maximizing the surfaces that can be evaluated during an investigation (8).

Sampling methods and number of samples are also influenced by the circumstances of the potential contamination. A sufficient number of samples must be taken to increase the probability that the sampling is representative, given the likely extent of contamination. In an initial investigation where a known or suspected release of potentially contaminated material has occurred, the first priority should be to collect samples near the suspected release source (often called directed or targeted sampling). In determining the extent of contamination, investigators should include coverage of areas along an anticipated contaminant pathway, i.e., those associated with air movement or dust collection, as well as activities that result in re-aerosolization or cross-contamination.

When sampling to identify contamination in a facility, the length of time between the suspected contamination of the facility and the time that sampling occurs is also important in determining where and how to collect samples. For example, since the sampling on November 11 was conducted >3 weeks after contamination was probably introduced into the facility, any aerosolized spores of *B.* *anthracis* had likely already settled on surfaces, and therefore surface sampling, as opposed to air sampling, was reasonable.

The environmental investigation did not identify anthrax spores in the patient’s home, possibly because her house was routinely cleaned thoroughly or because the piece of mail that was the source for her infection was not identified. One resident of her community is known to have received an envelope from which *B. anthracis* spores were isolated that was likely to have become cross-contaminated as it passed through the postal system, although no one in that household became ill (2). The patient also probably became ill following exposure to a low number of *B. anthracis* spores, which may explain why she had a relatively long incubation period compared with the other cases reported (9,10). Other host factors, including advanced age, underlying lung disease, medication use (2), and the practice of tearing up bulk mail (4), may have increased her chances of acquiring the disease.

The results of our investigation influenced the adherence and compliance of postal workers on postexposure prophylaxis at SCPDC. A study conducted there showed that 13% of the postal workers stopped taking postexposure prophylaxis because of the initial report of negative environmental cultures in the facility. An increase in postexposure prophylaxis adherence occurred, however, following the positive results in the facility (11).

The reasons why no postal workers at SCPDC became ill during this event are unknown. Perhaps host factors were important or anthrax spores were not aerosolized in sufficient concentration. The finding that spores were not widespread in the facility suggests that the dispersion was likely not due to substantial aerosolization. Following the experience from the Brentwood facility in October 2001, cleaning practices in postal facilities nationwide changed from use of compressed air, which easily aerosolized small particulate materials such as anthrax spores, to use of HEPA vacuums for cleaning (12). At SCPDC, maintenance workers stopped using forced air to clean equipment on October 27, 2001, which may have reduced the time when spores could have been aerosolized. The highly contaminated DBCS machine could have been a source of exposure to postal workers if the cleaning measures had not been changed.

The environmental investigation was central in demonstrating a possible source of infection for the case of inhalational anthrax in Connecticut. Our investigation showed that extensive sampling was required and that epidemiologic investigation was essential in identifying sites for sampling. None of the dry or wet swab samples were positive. For future investigations of large facilities, we recommend the use of wet wipes and vacuum. Further research is needed to clarify the sensitivity of the sampling and analytical methods for known or suspected *B. anthracis* and to develop clear algorithms for sampling if future investigations are needed. This investigation also demonstrated that illness associated with cross-contaminated mail is a rare but possible phenomenon.
